# Bell's-a-palsy

**Published:** 2010

**Authors:** Mahesh Pundlik Kate

**Affiliations:** Department of Neurology, Sri Chitra Tirunal Institute for Medical Sciences and Technology, Trivandrum, India. E-mail: maheshkate@yahoo.co.in

Oh! Thy beautiful, cometh to the door,

twisted is the face and tears on the floor.

One eye is crying, other is open and dry,

numb is the face , sudden , she prays why?

The world is tasteless, sounds so loud,

smile has disappeared, improvement is cloud.

Virus is at folly, or swollen is the nerve,

critiques are fighting, for this facial curve.

Is the nerve slow, is there malady within

How is the sugar , that keeps me thinking.

Steroids a little, a little pump and bow

the smile will be back within a week or so…….

